# Male Circumcision for Prevention of HIV Transmission: What the New Data Mean for HIV Prevention in the United States

**DOI:** 10.1371/journal.pmed.0040223

**Published:** 2007-07-24

**Authors:** Patrick S Sullivan, Peter H Kilmarx, Thomas A Peterman, Allan W Taylor, Allyn K Nakashima, Mary L Kamb, Lee Warner, Timothy D Mastro

## Abstract

Recent clinical trials in Africa found that male circumcision reduces the risk of acquiring HIV from heterosexual sex--what are the implications of these studies for the United States?

Three randomized, controlled clinical trials in South Africa, Kenya, and Uganda were recently unblinded early because interim analyses concluded that circumcision of HIV-negative adult males reduced their risk for acquiring HIV infection through penile–vaginal sex [1–3]. In each trial, men who had been randomly assigned to an intervention group receiving circumcision had a lower incidence of HIV infection in up to two years of follow up, compared to men who were assigned to a control group not receiving circumcision. The estimated reduction in the risk of HIV infection ranged from 51% to 60%; per-protocol estimates of risk reduction ranged from 55% to 76%.

It is now clear that male circumcision can be efficacious for men in reducing their risk of HIV acquisition through sex with women [4]. Some experts predict that the impact of male circumcision as a biomedical intervention for HIV prevention in Africa could be large [5,6], and preparatory work has been done to establish male circumcision programs in Africa. The implications of African trials on circumcision for HIV prevention programs in the United States are less clear—despite the interest of the popular press in the idea [7]. Here, we consider the differences between the HIV epidemics in Africa and the US, the current status of male circumcision in the US, and the knowledge gaps that will need to be addressed as we consider whether male circumcision should be evaluated or implemented as a biomedical intervention to reduce sexually acquired HIV infections domestically.

## Epidemiological Differences

The results of any trial must be interpreted with the caution that inference not be extended to populations differing from the study participants in important ways. The HIV epidemics in Africa are substantially different from the US epidemic. Generalized HIV epidemics exist in many areas of Africa, and the population prevalence of HIV among adult Kenyans, Ugandans, and South Africans ranges from 6%–19% [8]. The predominant mode of HIV transmission in Africa is male–female sex. In contrast, the US has a concentrated epidemic, with most sexual transmission occurring among men who have sex with men (MSM). The general population prevalence of HIV is about 0.4% in the US [9], and only 15% of men diagnosed with HIV infection during 2005 were reported to have acquired HIV through male–female sex [10].

## Biological Plausibility of Circumcision to Prevent HIV Acquisition

The association between circumcision and reduced risk for HIV acquisition is biologically plausible: the foreskin contains high concentrations of superficial Langerhans cells, CD4^+^ T cells, and macrophages [11]—all target cells for HIV infection, some of which may also be close to the skin surface [12,13]. In addition, the preputial sac may serve as a reservoir for HIV-containing secretions, resulting in prolonged contact time after exposure to secretions, and the foreskin may present less of a physical barrier to HIV entry than the more heavily keratinized skin of the shaft of the penis [12], and may have more frequent epithelial disruption. There are also potential indirect mechanisms of association between lack of circumcision and HIV risk; for example, lack of circumcision is associated with increased risk of genital ulcer diseases, which in turn are associated with increased risk of HIV transmission and acquisition [14].

## Considerations for Prevention of HIV Transmission by Penile–Vaginal Sex in the US

Epidemic differences are important because, on a population basis, the impact of circumcision as an intervention to prevent HIV infection among men who have sex with women will depend on the likelihood of HIV exposure among such men in the US—and, therefore, on the prevalence of HIV among their female sex partners. A recent analysis of data from sexually transmitted disease clinics in Baltimore evaluated the association of male circumcision and risk of prevalent HIV infection in two ways—first, evaluating all male attendees at the clinics, and second, restricting the analysis to males who were known to have been exposed to HIV heterosexually (e.g., sexual contacts of partners known to be infected with HIV) [15]. The results indicated that, while circumcision was not associated with lower HIV infection in the entire population of male STD clinic attendees, where HIV prevalence was 3%, circumcision was associated with significantly lower HIV prevalence in the subset of men with a known infected female sex partner, where the group's prevalence of infection was markedly higher at 12% (adjusted odds ratio [aOR] = 0.46; 95% confidence interval [CI] 0.22–0.97). In effect, this analysis illustrated the impact of partner prevalence of HIV on the association of circumcision and HIV infection status, and concluded that it was difficult to detect a protective effect from circumcision on HIV infection in the setting of a partner pool with lower HIV prevalence.

## Considerations for Prevention of HIV Transmission by Male–Male Sex

Most sexual transmission of HIV in the US occurs through male–male sex [10], most often infecting the receptive partner in penile–anal intercourse [16]. The results from the African trials demonstrated that circumcision was protective for men who were the insertive partner in vaginal intercourse, suggesting that the utility of male circumcision in preventing HIV transmission among MSM may be limited. Because reducing the concentration of target cells for HIV infection on the penis is a proposed protective mechanism, understanding the relative viral challenge presented by vaginal versus anal–rectal secretions is relevant to evaluating the plausibility of a protective effect of circumcision for the insertive male partner during anal intercourse. The concentration of HIV RNA in rectal secretions may be higher than in blood or semen, regardless of use of antiretroviral therapy [17], and may be orders of magnitude higher than the concentrations in vaginal or cervical secretions [17,18]. Circumcision may change the balance of virus and target cells, but if rectal mucosal secretions contain a higher concentration of infectious virus than vaginal secretions, any potential protective effect of circumcision for the insertive partner may be overwhelmed by excess virus. Also, new data suggest that, for limited periods of time before wound healing is complete, female sex partners of newly circumcised HIV-infected men may be at increased risk of acquiring HIV [4]. Possible transient increased risk of transmission (before complete wound healing) from recently circumcised HIV-infected MSM to their receptive anal intercourse partners would also be of concern.

Few studies provide evidence as to whether circumcision may protect against HIV infection among MSM. In a vaccine preparedness cohort of MSM followed from 1995 to 1997, circumcision was significantly associated with a decreased risk for HIV seroconversion (aOR = 0.5; 95% CI 0.3–0.9), controlling for number of male sex partners and unprotected sex with an HIV-positive partner [19]. In a cross-sectional survey of gay men in Seattle in the early 1990s, circumcision was associated with decreased odds of prevalent HIV infection (aOR = 0.5; 95% CI 0.25–1.0) [20]. While falling short of the quality of data from a randomized intervention trial, these limited data suggest that circumcised MSM in the US may have decreased risk of HIV infection. However, it is possible that the noted associations in these two observational studies were related to uncontrolled bias. A small cross-sectional study of Australian MSM found no association between circumcision status and risk of HIV infection, when stratifying by insertive and receptive roles [21].

## WHO/UNAIDS Technical Consultation on Male Circumcision and HIV Prevention: Research Implications for Policy and Programming

In March 2007, the World Health Organization and the Joint United Nations Programme on HIV/AIDS held a technical consultation on male circumcision and issued a summary document providing conclusions and recommendations relating to policy and programming on male circumcision and HIV prevention [4]. The document hails the results of the three African trials as “an important landmark in the history of HIV prevention” and states that male circumcision should be recognized as an efficacious intervention for the prevention of heterosexually acquired HIV infection in men. It was noted that male circumcision does not provide complete protection against HIV, and should always be considered as part of a comprehensive HIV prevention package. The document also concluded that the population level impact of male circumcision will be greatest in settings where the prevalence of heterosexually transmitted HIV infection is high, the levels of male circumcision are low, and populations at risk are large. Further, the document provides guidance about communication strategies, ethical and cultural issues, programmatic issues, financing issues, and needs for supporting health care services in developing countries. The document also explicitly states that, based on limited available data, promoting circumcision for HIV-positive men is not recommended. The full report of the technical consultation is available at: http://www.who.int/entity/hiv/mediacentre/MCrecommendations_en.pdf [4].

## Virological Issues

In the African countries where circumcision has been demonstrated to be efficacious, the predominant HIV subtypes are A, C, and D; it is likely that some recombinant strains were also represented in the Kenya and Uganda trials. In the US, subtype B predominates. Despite the theoretical possibility that subtype differences in either vaginal shedding of HIV or affinity to HIV receptors (especially those natively expressed on the foreskin) could modify the effectiveness of circumcision as an HIV prevention intervention, the consistent findings of the African trials argue that this is unlikely. For example, despite differences in vaginal shedding between subtype C and subtypes A and D [18], the efficacy of circumcision in trials where subtypes A, C, or D were prevalent was comparable. One potentially relevant biological difference relates to binding avidity of HIV subtypes for CCR5 receptors, which are important mechanisms for entry into Langerhans cells, and are the predominant HIV-1 co-receptor in foreskin immune cells [11]. Subtype C is reported to have lower binding avidity than subtype B for CCR5 receptors [22]; it is unclear whether the greater binding avidity of subtype B for CCR5 could represent an escape mechanism to overcome the decreased availability of target cells that results from circumcision.

## Status of Circumcision in the US

Public health recommendations will likely have the largest impact in populations where circumcision has been rare. Non-religious male circumcision was introduced to the US in the late 1800s [23], and by the 1940s, an increasing proportion of male children in the US were born in hospitals and were circumcised [24]. The proportion of newborns that were circumcised annually reached 80% after World War II and peaked in the mid-1960s. The proportion of male babies circumcised subsequently decreased. According to the National Hospital Discharge Survey, which documents circumcisions performed in hospitals but would not ascertain circumcisions performed outside of the hospital for religious reasons, 65% of newborns were circumcised in 1999. Although the overall proportion of newborns circumcised has been stable from 1979 to 1999 [25], the proportion of black newborns who were circumcised rose over this period to approximately 65%. Significant discrepancies also exist by region. While the proportion of newborns born in the Midwest who were circumcised increased over the 20-year period to 81% in 1999, the proportion of infants born in the West who were circumcised decreased over the same period, to 37% in 1999 [25].

Data from another hospital discharge survey, the National Inpatient Sample, present a slightly different picture [26]. In that survey, newborn circumcision rates increased from 48% in 1988–1991, to 61% in 1997–2000. Circumcision was more common among newborns born to families of higher socioeconomic status, in the Northeast or Midwest, and among newborns who were black [26].

Data from the National Health and Nutrition Examination Surveys from 1999 to 2004 indicated that the overall prevalence of circumcision among adult males in the US was 79% and varied by race/ethnicity (88% in non-Hispanic white men, 73% in non-Hispanic black men, 42% in Mexican Americans, and 50% in others). The prevalence of circumcision decreased among US-born men from the 1970s to the 1980s [27]. Although causality cannot be implied by these data and many other factors are likely operative, the rates of HIV and AIDS among non-Hispanic black and Hispanic men are considerably higher than in non-Hispanic white men in the US [28].

## Willingness of Adult Males to Be Circumcised

The ability of investigators to fully enroll three trials of adult circumcision [1–3] in Africa speaks to the acceptability of circumcision among adult males in South Africa, Kenya, and Uganda. A recent systematic review of published literature suggests that adult male circumcision may be acceptable as an HIV prevention intervention in many countries in sub-Saharan Africa [29]. In the US, the overwhelming majority of circumcisions are performed on newborns; adult circumcisions are commonly only done for medical reasons, such as preputial cancer or phimosis. It is not clear whether adult circumcision, were it to be recommended in the US, would be acceptable as a prevention intervention. Preliminary evidence from interviews with uncircumcised MSM surveyed at Gay Pride festivals in the US suggests that the majority of MSM would consider circumcision as an adult, if circumcision were shown to reduce risk of HIV infection by male–male sex [30]—although respondents were not told in the survey that protection would be partial or that condom use would still be recommended after circumcision.

## Policy Issues Related to Circumcision of Newborn Boys

The American Academy of Pediatrics changed from a less conclusive stance on circumcision in 1989 [31], which cited potential medical benefits and advantages (primarily reduced occurrence of phimosis and penile cancer) as well as disadvantages and risks, to their statement in 1999 that available data were not sufficient to recommend routine neonatal circumcision [32]. The 1999 position was re-affirmed in 2005 by the Academy after publication of the results of the South Africa trial [33]. In a 1995 US review, 61% of infant circumcisions were paid by private insurance, 36% were paid for by Medicaid, and 3% were self-paid by the parents of the infant [34]. Since 1999, 16 states have eliminated Medicaid payments for circumcisions that were not deemed medically necessary [35].

## Should Adult Male Circumcision Be Recommended for HIV Prevention in the US?

Circumcision may have a role for the prevention of HIV transmission in the US. However, because of the many differences between the underlying HIV epidemics in Africa and the US, differences in the prevalence of male circumcision in Africa and the US, and the considerable gaps in knowledge that exist regarding the potential impact of circumcision on HIV transmission by male–male sex, the extent of this role on a population basis is unknown. Further, the already high prevalence of circumcision among US men suggests some limitations in the potential impact of circumcision at a population level.

Based on the data from the three African clinical trials, it is likely that circumcision will decrease the probability of a man acquiring HIV via penile–vaginal sex with an HIV-infected woman in the US. Until public health recommendations are available for the US, some sexually active men may consider circumcision as an additional HIV prevention measure, but should do so only in consultation with their physician or health care provider, and with a clear understanding of the costs and risks of circumcision and the need to continue use of other, proven prevention measures (e.g., reducing the numbers of sex partners and using condoms consistently and correctly). Men who choose to be circumcised should also be counseled about the importance of refraining from sexual intercourse following circumcision, until wound healing is complete [4].

To consider the possible impact of public health recommendations for male circumcision, we must also take into account HIV incidence in high-risk groups, as well as adoption of other protective behaviors, such as condom use. For example, HIV incidence among US MSM recruited in community- and venue-based samples was, on average, about 1.9% annually [36], and 36% of MSM in the US National HIV Behavioral Surveillance System reported having unprotected anal sex with a casual partner in the last 12 months before interview [37]. There are few data on HIV incidence among high-risk heterosexuals in the US, but there are limited data on condom use: in 2002, 16% of high-risk heterosexual men and 24% of high-risk heterosexual women reported that they never used condoms during penile–vaginal sex with a non-primary partner [38]. Currently available data on disparities in rates of prevalent HIV infection and AIDS [28,39] and the prevalence of circumcision among US men suggest that black and Hispanic men may have particular opportunities for reduction of risk of HIV acquisition through circumcision.

## Future Research and Consultation

In order to understand the potential for male circumcision as an HIV prevention approach in the US, we believe that there are important questions that should be answered. These include questions that can be answered by basic science, by modeling, by surveys of acceptability, by considering ethical issues, and, perhaps, by clinical trials in the US. For example, it is important to understand more fully the differences in shedding of HIV by rectal versus vaginal mucosa. Modeling may provide important information on (1) the impact on the US epidemic from increasing male circumcision rates, and (2) the cost–benefit ratio of circumcision among newborns, or among adult men with high risk of exposure to HIV through sex. Cost–benefit models may be limited by lack of definitive transmission parameters in US populations and should therefore be conducted with appropriate sensitivity analyses. Surveys may increase our understanding of the acceptability of adult male circumcision among groups of uncircumcised adult males in the US for whom circumcision might be recommended (e.g., men who have unprotected vaginal or anal intercourse with HIV-infected partners, or with multiple partners of unknown serostatus), and of barriers and facilitators to acceptance of adult male circumcision, were it recommended as an HIV prevention strategy. Given recent trial results and international consensus that male circumcision is efficacious, it is important to consider ethical questions about whether equipoise exists for a US MSM trial, and about how to implement trials or programs of male circumcision in the context of complex cultural and religious views about circumcision [27]. Evaluating data from basic science, modeling, and acceptability surveys and addressing ethics questions will be important in deciding whether a clinical trial to determine the efficacy of male circumcision among MSM may be feasible and appropriate in the US.

Further, recommendations about circumcision in newborns or high-risk adults for the prevention of HIV infection cannot be made without a more comprehensive discussion of other, documented disease prevention benefits and risks of circumcision. Benefits include reduction in acquisition of sexually transmitted genital ulcer disease, infant urinary tract infections, penile cancer, and cervical cancer in female sex partners [14,40–43]. Although this is less clear, circumcision may also be associated with reduced risk of herpes simplex virus 2 infection [14]. Risks include postoperative infection, damage to the penis, excessive bleeding, problems with postoperative appearance of the penis, and anesthesia-related problems [1–3,40]. If it is determined that circumcision can play a role in preventing HIV transmission and other adverse health outcomes in the US, it will be important to consider the extent to which circumcision is included in public and private medical insurance benefits. The cost, medical risks, and potential benefits of circumcision for HIV prevention will need to be considered separately for infants, high-risk heterosexuals, and high-risk MSM. Relatively high rates of circumcision have prevailed in the US, where rates of HIV infection are currently relatively low. To the extent that a high prevalence of circumcision may have hypothetically led to lower HIV rates in the US, reducing reimbursement and declining rates of the procedure could reverse this beneficial effect.

To address these scientific and policy questions with a broad group of stakeholders, the US Centers for Disease Control and Prevention convened a consultation in April 2007 to gain diverse input about the public health research agenda and to develop public health recommendations about the role of male circumcision for prevention of HIV in the US. The summary of the outcomes of the consultation will be made available later in 2007, via the Centers' Division of HIV/AIDS Prevention Web site (http://www.cdc.gov/hiv/).

## Abbreviations

aOR: adjusted odds ratio
CI: confidence interval
MSM: men who have sex with men

## References

[pmed-0040223-b001] Auvert B, Taljaard D, Lagarde E, Sobngwi-Tambekou J, Sitta R (2005). Randomized, controlled intervention trial of male circumcision for reduction of HIV infection risk: The ANRS 1265 trial. PLoS Med.

[pmed-0040223-b002] Bailey RC, Moses S, Parker CB, Agot K, Maclean I (2007). Male circumcision for HIV prevention in young men in Kisumu, Kenya: A randomised controlled trial. Lancet.

[pmed-0040223-b003] Gray RH, Kigozi G, Serwadda D, Makumbi F, Watya S (2007). Male circumcision for HIV prevention in men in Rakai, Uganda: A randomised trial. Lancet.

[pmed-0040223-b004] Joint United Nations Programme on HIV/AIDS (2007). New data on male circumcision and HIV prevention: Policy and programme implications.

[pmed-0040223-b005] Williams BG, Lloyd-Smith JO, Gouws E, Hankins C, Getz WM (2006). The potential impact of male circumcision on HIV in sub-Saharan Africa. PLoS Med.

[pmed-0040223-b006] Gray RH, Li X, Kigozi G, Serwadda D, Nalugoda F (2007). The impact of male circumcision on HIV incidence and cost per infection prevented: A stochastic simulation model from Rakai, Uganda. AIDS.

[pmed-0040223-b007] Smith S (2006 October 16). Circumcision may help fight AIDS in Africa, but in the US, the medical argument is iffy.

[pmed-0040223-b008] Joint United Nations Programme on HIV/AIDS, World Health Organization (2006). AIDS epidemic update.

[pmed-0040223-b009] McQuillan GM, Kruszon-Moran D, Kottiri BJ, Kamimoto LA, Lam L (2006). Prevalence of HIV in the US household population: The National Health and Nutrition Examination Surveys, 1988 to 2002. J Acquir Immune Defic Syndr.

[pmed-0040223-b010] US Centers for Disease Control and Prevention (2007). HIV/AIDS surveillance report: Cases of HIV infection and AIDS in the United States and dependent areas, 2005.

[pmed-0040223-b011] Patterson BK, Landay A, Siegel JN, Flener Z, Pessis D (2002). Susceptibility to human immunodeficiency virus-1 infection of human foreskin and cervical tissue grown in explant culture. Am J Pathol.

[pmed-0040223-b012] McCoombe SG, Short RV (2006). Potential HIV-1 target cells in the human penis. AIDS.

[pmed-0040223-b013] Donoval BA, Landay AL, Moses S, Agot K, Ndinya-Achola JO (2006). HIV-1 target cells in foreskins of African men with varying histories of sexually transmitted infections. Am J Clin Pathol.

[pmed-0040223-b014] Weiss HA, Thomas SL, Munabi SK, Hayes RJ (2006). Male circumcision and risk of syphilis, chancroid, and genital herpes: A systematic review and meta-analysis. Sex Transm Infect.

[pmed-0040223-b015] Warner L, Ghanem KG, Newman D, Macaluso M, Sullivan P (2006). Male circumcision and risk of HIV infection among heterosexual men attending Baltimore STD clinics: An evaluation of clinic-based data [presentation].

[pmed-0040223-b016] Varghese B, Maher JE, Peterman TA, Branson BM, Steketee RW (2002). Reducing the risk of sexual HIV transmission: Quantifying the per-act risk for HIV on the basis of choice of partner, sex act, and condom use. Sex Transm Dis.

[pmed-0040223-b017] Zuckerman RA, Whittington WL, Celum CL, Collis TK, Lucchetti AJ (2004). Higher concentration of HIV RNA in rectal mucosa secretions than in blood and seminal plasma, among men who have sex with men, independent of antiretroviral therapy. J Infect Dis.

[pmed-0040223-b018] John-Stewart GC, Nduati RW, Rousseau CM, Mbori-Ngacha DA, Richardson BA (2005). Subtype C is associated with increased vaginal shedding of HIV-1. J Infect Dis.

[pmed-0040223-b019] Buchbinder SP, Vittinghoff E, Heagerty PJ, Celum CL, Seage GR (2005). Sexual risk, nitrite inhalant use, and lack of circumcision associated with HIV seroconversion in men who have sex with men in the United States. J Acquir Immune Defic Syndr.

[pmed-0040223-b020] Kreiss JK, Hopkins SG (1993). The association between circumcision status and human immunodeficiency virus infection among homosexual men. J Infect Dis.

[pmed-0040223-b021] Grulich AE, Hendry O, Clark E, Kippax S, Kaldor JM (2001). Circumcision and male-to-male sexual transmission of HIV. AIDS.

[pmed-0040223-b022] Marozsan AJ, Moore DM, Lobritz MA, Fraundorf E, Abraha A (2005). Differences in the fitness of two diverse wild-type human immunodeficiency virus type 1 isolates are related to the efficiency of cell binding and entry. J Virol.

[pmed-0040223-b023] Schoen E (2005). On circumcision.

[pmed-0040223-b024] Laumann EO, Masi CM, Zuckerman EW (1997). Circumcision in the United States. Prevalence, prophylactic effects, and sexual practice. JAMA.

[pmed-0040223-b025] US Centers for Disease Control and Prevention (2000). Trends in circumcision among newborns.

[pmed-0040223-b026] Nelson CP, Dunn R, Wan J, Wei JT (2005). The increasing incidence of newborn circumcision: Data from the nationwide inpatient sample. J Urol.

[pmed-0040223-b027] Xu F, Markowitz LE, Sternberg MR, Aral SO (2007). Prevalence of circumcision and herpes simplex virus type 2 infection in men in the United States: The National Health and Nutrition Examination Survey (NHANES), 1999–2004. Sex Transm Dis.

[pmed-0040223-b028] US Centers for Disease Control and Prevention (2007). Racial/ethnic disparities in diagnoses of HIV/AIDS—33 states, 2001–2005. MMWR Morb Mortal Wkly Rep.

[pmed-0040223-b029] Westercamp N, Bailey RC (2007). Acceptability of male circumcision for prevention of HIV/AIDS in sub-Saharan Africa: A review. AIDS Behav.

[pmed-0040223-b030] Begley E, Jafa K, Voetsch A, Heffelfinger J, Sullivan PS (2007). Willingness of men who have sex with men in the US to be circumcised as adults to reduce risk of HIV infection [abstract 983].

[pmed-0040223-b031] [No authors listed] (1989). American Academy of Pediatrics. Report of the Task Force on Circumcision. Pediatrics.

[pmed-0040223-b032] [No authors listed] (1999). American Academy of Pediatrics. Circumcision policy statement. Pediatrics.

[pmed-0040223-b033] [No authors listed] (2005). American Academy of Pediatrics. AAP publications retired and reaffirmed. Pediatrics.

[pmed-0040223-b034] Mansfield CJ, Hueston WJ, Rudy M (1995). Neonatal circumcision: Associated factors and length of hospital stay. J Fam Pract.

[pmed-0040223-b035] National Conference of State Legislatures (2006). State health notes: Circumcision and infection.

[pmed-0040223-b036] Stall RD (2006). Re-emerging HIV epidemics among MSM in the United States and other industrialized nations: Evidence and insight [abstract THBS0202].

[pmed-0040223-b037] Sanchez T, Finlayson T, Drake A, Behel S, Cribbin M (2006). Human immunodeficiency virus (HIV) risk, prevention, and testing behaviors—United States, National HIV Behavioral Surveillance System: Men who have sex with men, November 2003–April 2005. MMWR Surveill Summ.

[pmed-0040223-b038] US Centers for Disease Control and Prevention (2004). HIV testing survey, 2002. HIV/AIDS special surveillance report 5.

[pmed-0040223-b039] Espinoza L, Hall HI, Hardnett F, Selik RM, Ling Q (2007). Characteristics of persons with heterosexually acquired HIV infection, United States 1999–2004. Am J Public Health.

[pmed-0040223-b040] Moses S, Bailey RC, Ronald AR (1998). Male circumcision: Assessment of health benefits and risks. Sex Transm Infect.

[pmed-0040223-b041] Alanis MC, Lucidi RS (2004). Neonatal circumcision: A review of the world's oldest and most controversial operation. Obstet Gynecol Surv.

[pmed-0040223-b042] Fergusson DM, Boden JM, Horwood LJ (2006). Circumcision status and risk of sexually transmitted infection in young adult males: An analysis of a longitudinal birth cohort. Pediatrics.

[pmed-0040223-b043] Diseker RA, Peterman TA, Kamb ML, Kent C, Zenilman JM (2000). Circumcision and STD in the United States: Cross sectional and cohort analyses. Sex Transm Infect.

